# Preference of Dental Practitioners toward the Use of Local and Topical Anesthetics for Pediatric Patients in Saudi Arabia: A Cross-Sectional Survey

**DOI:** 10.3390/children8110978

**Published:** 2021-10-28

**Authors:** Fahad Shulaywih Alanazi, Mohammed Fahad Alhazzaa, Yazeed Mohammed Alosaimi, Faisal Abdullah Alajaji, Atallah Shulaywih Alanazi, Abdullah Alassaf, Basim Almulhim, Sara Ayid Alghamdi, Sreekanth Kumar Mallineni

**Affiliations:** 1 College of Dentistry, Majmaah University, Almajmaah 11952, Saudi Arabia; F_oj1@hotmail.com (F.S.A.); Mf.alhazzaa@gmail.com (M.F.A.); Yazeed-2mo@hotmail.com (Y.M.A.); Fi9aal.9@gmail.com (F.A.A.); 2 Humeidan and Khowaiter, Riyadh 12631, Saudi Arabia; Atallah16@hotmail.com; 3 Department of Preventive Science, College of Dentistry, Majmaah University, Almajmaah 11952, Saudi Arabia; am.assaf@mu.edu.sa (A.A.); b.almulhim@mu.edu.sa (B.A.); Sa.mohammed@mu.edu.sa (S.A.A.); 4 Department of Pediatric and Preventive Dentistry, Saveetha Dental College and Hospital, Saveetha Institute of Medical and Technical Sciences, Saveetha University, Chennai 600077, India

**Keywords:** local anesthesia, topical anesthesia, children, dentists

## Abstract

Background: Local anesthesia administration techniques are slightly challenging to perform and master on the basis of experience. It is always delicate to adjust to the first patient injection, especially in children. This study investigated dental practitioners’ preferences toward topical and local anesthetics for children in Saudi Arabia. Materials and methods: A questionnaire was sent through google forms to the participants, including sociodemographic characteristics, as well as perceptions of local anesthesia and topical anesthesia. The details for each demographic variable were based the gender, occupation, and experience years. Descriptive statistics were carried out using SPSS (version 24.0), where a *p*-value of 0.05 at a 95% confidence interval was considered significant. Result: A total of 274 responses were received from Saudi Arabian dental practitioners, whereby 92.3% preferred lidocaine, and, while choosing local anesthesia, most participants (57.7%) considered precise bodyweight. The majority of the participants selected 27 gauge needles for infiltrations (46.3%) and blocks (63.9%). Short needles were preferred by the majority (93.4%) of the dental practitioners for infiltration, while long needles (83.9%) were chosen for nerve blocks. Benzocaine (68.2%) was preferred by the majority of the dental practitioners for topical anesthesia, and 55.8% of them were not aware of the brand of the topical anesthesia. The majority of dental practitioners felt that topical anesthesia was effective prior to administration of local anesthesia, and 83.6% of the Arabian dental practitioners expressed that patients complained regarding the taste of topical anesthesia. There were mixed opinions observed among the genders and occupations of dental practitioners regarding anesthetics used. Conclusion: The dental practitioners’ perceptions and preferences demonstrate that the most commonly preferred type of local anesthetic was lidocaine, whereas the most preferred type of topical anesthetic was benzocaine in gel form. Moreover, the most widely used factor in deciding the dosage of local anesthesia was precise body weight among Saudi dentists. The majority of participants preferred short needles for infiltrations and long needles for nerve blocks. The 27 gauge needle was chosen by the majority of the participants for both infiltration and nerve blocks.

## 1. Introduction

Anesthesia is a vital part of clinical Dentistry. It offers painless care and helps a dentist operate correctly within optimum comfort [1,2]. Painful dental treatments induce fear, while fear and anxiety increase the pain sensed [1]. In children, local anesthesia administration leads to fear and anxiety and is associated with pain and discomfort [1,2]. There is a close connection between a child’s dental anxiety and pain. Injection administration of local anesthesia is still the most common approach used in Dentistry [3]. Topical local anesthetic agents are applied to minimize the pain that results from the injection [4,5,6]. Topical anesthetics reduce the discomfort of needle penetration, and topical agents are available as a gel, liquid, spray, ointment, and patch [6]. The most widely used topical anesthesia is benzocaine, and it has a rapid-onset action. Topical anesthesia agents are applied prior to the local anesthesia administration site to minimize pain [4,6]. Nonetheless, after prolonged or repetitive use, localized allergic reactions can occur. Moreover, lidocaine and compound topical anesthetics have an issue of risk of methemoglobinemia [6].

Local anesthesia is the best and most successful approach to dental pain relief. Many local anesthetic agents are available to minimize pain and discomfort [7]. Children would typically have an adverse reaction to dental anesthesia, including a lack of cooperation with dental therapy and hysteric activity, making it impossible for the dentist to administer the procedure correctly [8]. Nonetheless, not all children are affected by actions, as dental fear is mainly due to a sense of loss of control and pain perception that may emerge from a child’s awareness of teeth and past painful suffering [8,9]. Local anesthesia administration techniques are slightly challenging to perform and master on the basis of experience. It is also challenging to deal with pediatric patients in dental operations while administering local anesthesia [3,9]. It is essential to regularly assess the perceptions and practices among dental practitioners. There is value in determining the perceptions of topical and local anesthetics in children among Saudi Arabian dental practitioners, and no studies have been published on this area. Therefore, this study aimed to investigate dental practitioners’ preferences of topical and local anesthesia to assess current practices in Saudi Arabia.

## 2. Materials and Methods

This cross-sectional questionnaire-based study was carried out following the STROBE guidelines. The ethical approval was obtained from the Institution Ethical Committee, Majmaah University, Saudi Arabia (IRB No: MUREC-Feb-28/Com-2021/24-2). The study was conducted among Saudi Arabian dental practitioners to investigate their perceptions and practices toward using local anesthesia and topical anesthesia for a period of 5 months from 1 February to 30 June 2021. A cross-sectional survey was sent through Google Forms to the participants. The questionnaire included 17 questions: seven questions for local anesthesia and 10 for topical anesthesia with respect to dental practitioners’ preferences, distributed through social media, general dentists, specialists, and consultants. The questionnaire was adopted from Kohli et al. [10] from a survey performed among dental practitioners in the United States of America. The survey included questions related to the most commonly used local and topical anesthetics drugs, as well as the most critical factor in deciding the dosage of local anesthesia. The questionnaire also had the most frequently used needle length and gauge for infiltrations and blocks, the adverse drug reaction to anesthetics, and the length of time to infuse a complete cartridge. The commonly used type and form of topical anesthesia, the effectiveness, and a common complaint from the patient regarding topical anesthetics were included in the questionnaire. Demographic data including gender and education were collected. The comparisons were made on the basis of gender (male and female) and occupation (consultants, general practitioners, and specialists). The data were analyzed using the statistical package IBM SPSS Statistics for Windows (Version 24.0. Armonk, NY, USA: IBM Corp; 2016) by computing the percentage response for each question. The comparisons were made in percentages using the chi-square test, with a *p*-value less than 0.05 considered significant.

## 3. Results

A total of 274 dental participants responded to the questionnaire in the study. The distribution of the participants was based on gender and occupation. Most of the participants were females (62.8%), with males accounting for 37.2%. In the study population, the majority of subjects were general practitioners (83.9%), with the remainder consisting of specialists (13.2%) and consultants (2.9%). The most commonly used local anesthetic drug was lignocaine (92.3%) (Table 1); 86% of female and 81.6% of male practitioners preferred using lignocaine (Table 2), and the findings were not statistically significant (*p* > 0.05). The majority of the consultants (100%), general practitioners (91.7%), and specialists (94.4%) also preferred using lignocaine as a local anesthetic drug (Table 2). The findings were not statistically significant (*p* > 0.05). The essential factor considered when deciding on the dose of the local anesthetic administration was body weight (57.7%), preferred by both males (56.9%) and females (58.1%), and the findings were not statistically significant (*p* > 0.05). Furthermore, 50% of the consultants preferred age as the most common factor for deciding the dose of the local anesthetic drug, while general practitioners (58.3%) and specialists (61.1%) preferred body weight as an essential factor. The findings were not statistically significant (*p* > 0.05). Most Arabian dental practitioners preferred 27 gauge needles for infiltration and nerve blocks in children (Table 1). Forty-eight percent of males and 45.3% of females preferred 27 gauge needles for infiltration, while 68% of females and 56.9% of males preferred 27 gauge needles for nerve blocks in children (Table 2). The findings were not statistically significant (*p* > 0.05). In the case of infiltration, the majority of consultants (62.5%) preferred 25 gauge needles, while general dental practitioners (47.4%) and specialists (50%) preferred 27 gauge needles (Table 3) for infiltrations in children. The findings were statistically significant (*p* > 0.05). In the case of nerve blocks, the majority of consultants (62.5%) also preferred 25 gauge needles, while general dental practitioners (66.5%) and specialists (61.1%) preferred 27 gauge needles (Table 3); the results were statistically significant (*p* < 0.05). In the case of the length of needles, 93.4% of the total Arabian dental practitioners preferred short needles for infiltration to long (2.9%) and ultra-short needles (3.6%), while 83.2% of them preferred long needles for nerve blocks in children (Table 1). Both males (95.3%) and females (90.2%) preferred short needles (Table 2), and the results were not statistically significant (*p* < 0.05). The majority of the consultants (50%), general dental practitioners (93.9%), and specialists (100%) preferred short needles in children for infiltrations (Table 3), and the results were statistically significant (*p* < 0.05).

In the case of nerve blocks, the majority of the males (74.5%) and females (88.4%) preferred long needles (Table 2), and the results were statistically significant (*p* < 0.05). The majority of the consultants (87.5%), general dental practitioners (87.4%), and specialists (55.6%) preferred long needles in children for nerve blocks (Table 3), and the results were statistically significant (*p* < 0.05). Thirty-one percent of the Arabian dental practitioners opined that 11–20 s was required for administration of a full cartridge of a local anesthesia solution. The majority of females (31.4%) identified 11–20 s as ideal to inject a full cartridge of the local anesthetic solution, while the majority of males (30.4%) preferred 30–60 s (Table 2), and the results were statistically significant (*p* < 0.05). There was an evident disagreement among the consultants (>10 s, 37.5%), general dental practitioners (11–20 s, 34.3%), and specialists (31–60 s, 41.7%) regarding time to inject a full cartridge of local anesthetic solution (Table 3), and the results were statistically significant (*p* < 0.05). The overall scores achieved by Saudi Arabia dentists related to topical anesthetics used in children are summarized in Table 4. The majority of dental practitioners in Saudi Arabia (65.7%) often use topical anesthesia prior to local anesthesia (Table 4), with more females (68.6%) than males (60.8%) implementing this practice (*p* > 0.05) (Table 5). The most commonly used form of topical anesthesia was gel by both males (86.4%) and females (90.4%), and the findings were not statistically significant (*p* > 0.05). The most commonly used drug for topical anesthesia was benzocaine (females, 62.3% and males, 61.0%); the findings were not statistically significant (*p* > 0.05).

There was evident agreement among the Arabian dentists regarding the use of topical anesthesia in children prior to administration of local anesthesia: consultants (62.5%), general dental practitioners (65.2%), and specialists (69.4%) (Table 6). The results were statistically significant (*p* < 0.05). The majority of the consultants (100%), general dental practitioners (89.1%), and specialists (80.6%) preferred using a gel form of topical anesthesia prior to administration of local anesthesia in children (Table 6), and the results were statistically significant (*p* < 0.05). Sixty-eight percent of the dental practitioners (females 68.6%, males 67.6%; *p* > 0.05) opined that they preferred using benzocaine as topical anesthesia in children. The majority of the consultants (62.5%), general dental practitioners (68.3%), and specialists (69.4%) used benzocaine as topical anesthesia (*p* > 0.05).

On the question regarding the brand of topical anesthesia, 55.8% of Arabian practitioners responded that they were not aware of the brand. Among them, 57.65% of females and 52.9% of males were unaware of the topical anesthesia brand (Table 5). All the consultants (100%) who participated in the study were aware of the topical anesthesia brand. In comparison, general dental practitioners (57.4%) and specialists (58.3%) were not aware of the brand that they used for topical anesthesia (Table 6), and the results were statistically significant (*p* < 0.05). The female (43.6%) and male (53.9%) practitioners felt that more than 30 s was adequate for application of a topical anesthetic drug before administration of local anesthesia (Table 5), and the results were statistically significant (*p* < 0.05). The consultants (62.5%) felt that 21–30 s was ideal for application of topical anesthesia, while 33% of specialists preferred 31–60 s and 23% of general practitioners preferred more than 60 s (*p* > 0.05). More than one-third of dental practitioners (38.7%) felt that topical anesthesia is effective in children. Among them, 41.3% of females considered it effective, while 39.2% of males considered it very effective, and the findings were not statistically significant (*p* > 0.05). The majority of general dental practitioners (40.4%) and specialists (30.6%) felt that topical anesthesia application in children was effective, while 75% of consultants felt it was very effective (*p* < 0.05). The most common complaint of pediatric patients in dental operations regarding topical anesthesia was taste (83.6%). Among them, 87.8% of females and 76.5% of males reported taste as the most common complaint regarding topical anesthesia (*p* > 0.05). The majority of consultants (87.55), general dentists (83.5%), and specialists (88.9%) reported it as the most common complaint from children regarding topical anesthesia, and the results were statistically significant. For the question “How many of your patients experienced any drug side-effect to the topical anesthetic preparation in the last year?”, 82.8% of the Arabian dental practitioners reported no side-effects of topical anesthesia (females 86%; males 77.5%; *p* > 0.05). More than 50% of consultants observed side-effects with topical anesthesia, while 83% of general practitioners and 88.9% of specialists never encountered any adverse effects in children (Table 6). For the question on what percentage of the time topical anesthetics work when applied prior to the local anesthetic injection, 36.1% of the Arabian dental practitioners successfully used topical anesthesia in 75% of cases (Table 4). The majority of females (34.9%) and males (38.2%) expressed that they successfully used topical anesthesia in 75% of the cases (Table 5), and the findings were not statistically significant (*p* > 0.05). Fifty percent of the consultants reported successfully using topical anesthesia in 50% of cases, while 36.5% of general dentists and 36.1% of specialists reported successfully using topical anesthetics in 75% of cases (Table 6). The findings were statistically significant (*p* < 0.05).

## 4. Discussion

It is a big challenge in the pediatric population to distinguish behavior due to pain from behavior related to distress or fear associated with a mixture of environmental, social, parental, or developmental factors [11]. The behavior of the child in discriminating pain due to local anesthesia administration could determine whether patients make return appointments because children and their parents perceive a successful visit in delivering this essential component of care, or whether they find someone else whom they feel will be more sympathetic to their needs [12]. The present cross-sectional survey was accomplished to evaluate the perceptions and practices of local and topical anesthesia in children. A sample of 274 dental practitioners around Saudi Arabia was involved in the present study. The present survey was adopted from the research in the United States performed by Kohli et al. [10]. The questionnaire included commonly used local and topical anesthesia, commonly used needle gauge for infiltration and nerve blocks, widely used form of topical anesthesia, duration of application of local and topical anesthesia, and adverse effects experienced while administering anesthetic drugs.

The present study observed that lidocaine is the commonly used local anesthetic drug. Similar findings were reported by Kholi et al. [10] in the United States of America among pediatric dentists. A Canadian survey [13] also reported the frequent use of lidocaine 2% with epinephrine 1:100,000 for local anesthesia administration. Similar findings were reported by Khalil [14] from Saudi Arabia, who revealed that lidocaine is a commonly used local anesthetic drug in children. Among the study sample, 72% of general dentists and 74% of specialists preferred using lidocaine. However, in the present study, general practitioners (91.7%) and specialists (94.4%) also preferred using lignocaine as a local anesthetic drug; the findings were not in agreement with prior Saudi Arabian studies [14].

In the case of infiltration and nerve blocks in children, most Saudi dental practitioners preferred 27 gauge needles. The majority of the consultants (62.5%) preferred 25 gauge needles for infiltration in children, while general dental practitioners (47.4%) and specialists (50%) preferred 27 gauge needles. In the case of nerve blocks, the majority of consultants (62.5%) also preferred 25 gauge needles, while general dental practitioners (66.5%) and specialists (61.1%) preferred 27 gauge needles. The findings from the present study were in agreement with Kohli et al. study [10]. The present survey showed that 86.5% of practitioners use a long needle for nerve block and 91.9% use a short needle for infiltration, as recommended by Malamed [15], who proposed that a long needle should be used for all techniques requiring penetration of a significant thickness of soft tissue, whereas short needles should be used for penetration of insignificant depths of soft tissue. Moreover, Ram et al. [16] reported that children experienced pleasant mandibular block administered with a 27 gauge needle compared to a 30 gauge needle. No difference was evident in the maxillary infiltration provided with 27 or 30 gauge needles.

The primary aim of using local anesthesia is to treat the patient least painfully. This aim becomes particularly important when considering a very young, anxious, fearful, and/or needle-phobic patient. Numerous dental procedures may require no local anesthesia but may still have the potential for soft-tissue stimulation or pain. When used appropriately, adequate topical anesthesia can provide a safe and positive treatment outcome, improving patient behaviors and attitudes toward future care. The academic programs show some similarities across the entire kingdom of Saudi Arabia. Although benzocaine is the most used topical anesthesia, most dental practitioners in Saudi Arabia use topical anesthesia before local anesthesia administration. The clinical effectiveness of lidocaine and benzocaine has already been established [17]. Dentists who experience stress during the administration of mandibular blocks attach greater credibility to the child’s report of pain [14], and most patients’ determining factor for acceptance of topical anesthesia is taste [18,19]. Garg et al. [20] evaluated the efficacy of 2% lidocaine gel and 20% benzocaine gel for topical anesthesia. The authors used a split-mouth study design to compare these two topical anesthetic gels with placebo paste in the same patient for 1 min before needle insertion and found both are equally effective. However, in the present study, most dental practitioners preferred using benzocaine as a topical anesthesia agent. These findings are in agreement with the American study [10]. Only 37.9% of participants in this survey were willing to use a different topical anesthesia delivery system if available on the market, due to the widespread use of anesthetics, changes in injection technique, changes in anesthetic choice because of cost, and short-term changes in drug choice related to in-house availability [21]. Side-effects caused by topical anesthetics are not frequently reported by dental practitioners [20,22,23]. Pain at the injection site, damage of the periosteum, soft tissues, nerves, and blood vessels, needle breakage, and hypersensitivity reactions are commonly encountered complications with local anesthesia administration [7,22,23]. In the present survey, there were only questions about adverse effects in association with topical anesthesia. The majority of Arabian dental practitioners reported no side-effects of topical anesthesia; more than 50% of consultants experienced side-effects with topical anesthesia, while more than 80% of general practitioners specialists never encountered any adverse effects in children.

In earlier studies, Kohli et al. [10] surveyed pediatric dentists from the United States, a Canadian survey [13] was performed with the Royal College of Dental Surgeons of Ontario, and a prior study from Saudi Arabia surveyed 431 dentists (219 general practitioners and 212 specialists). In the present survey, 274 Arabian dental practitioners (230 general practitioners, 36 specialists, and eight consultants) participated. The survey was conducted through social media; hence, the response rate was not sought. This was considered a limitation of the study. The present study was conducted among general dental practitioners, consultants, and specialists, and the survey covered all dental professionals in Saudi Arabia. The comparisons among general dental practitioners, consultants, and specialists may not be feasible; hence, this was also considered a limitation. The consultants were significantly lower in number, which was also a possible limitation of the study. The province of the participant was not contemplated in data collection, which was also a potential limitation. The generalization of the findings is impossible, but these findings can be viewed as a reference for upcoming studies. Furthermore, a prospective study with a large sample size with an equal distribution of dentists from all provinces of Saudi Arabia is warranted. The present cross-sectional survey compared general dental practitioners, specialists, and consultants on the basis of gender and occupation setting. Moreover, the current survey is the only study to explore Arabian dental practitioners’ perceptions and preferences regarding local and topical anesthetics in children.

## 5. Conclusions

There were evident mixed opinions among genders and occupations of dental practitioners regarding topical and local anesthetics in Saudi Arabia. The most commonly preferred local anesthetic drug was lidocaine, and the most preferred topical anesthetic was benzocaine in gel form. The most widely used factor in deciding the dosage of local anesthesia was precise body weight among Saudi dentists. The 27 gauge needle was chosen by the majority of participants for both infiltration and nerve blocks. The majority of participants preferred short needles for infiltrations and long needles for nerve blocks. There was evidence of mixed opinions among genders and occupations of dentists in Saudi Arabia regarding anesthetic drugs in children.

## Figures and Tables

**Table 1:** Overall achieved scores by all the participants regarding local anesthetics used for children.

Questions	Responses
**What kind of local anesthesia do you most commonly use?**	
Articaine	1.5%
Bupivacaine	0.7%
Lidocaine	92.3%
Mepivacaine	4.4%
Prilocaine	1.1%
**When deciding on the dose of the local anesthetic you administer, what is the most important thing you consider?**	
Age and weight	0.4%
Duration of treatment	10.6%
Estimated child size	2.2%
Medical history	0.7%
One cartridge is given for all patients	6.2%
Patient’s medical status	0.4%
Precise age in years	8.8%
Precise body weight	57.7%
The level of difficulty of the procedure	12.8%
Weight and child size and level of difficulty	0.4%
**What needle gauge do you use most of the time for infiltration?**	
25 gauge	35.4%
27 gauge	46.4%
30 gauge	17.2%
Other	1%
**What needle gauge do you use most of the time for blocks?**	
25 gauge	21.9%
27 gauge	63.9%
30 gauge	13.1%
Other	1.2%
**What length of needle do you use most of the time for infiltration?**	
Long	2.9%
Short	93.4%
Ultrashort	3.6%
Other	0
**What length of needle do you use most of the time for blocks?**	
Long	83.2%
Short	16.4%
Ultrashort	0.4%
Other	0%
**How long does it take you to inject a full cartridge of a local anesthetic solution?**	
10 s or less	8.8%
11–20 s	31.4%
21–30 s	25.9%
31–60 s	27.0%
61 s or more	6.9%

Detailed questions are listed in bold.

**Table 2:** Perceptions of local anesthetics among dental practitioners based on gender.

Questions	Responses	Female	Male	*p*-Value
What kind of local anesthesia do you most commonly use?	Articaine	1.7%	1.0%	0.06
	Bupivacaine	0.6%	1.0%	
	Lidocaine	91.3%	94.1%	
	Mepivacaine	4.7%	3.9%	
	Prilocaine	1.7%	0.0%	
When deciding on the dose of the local anesthetic you administer, what is the most important thing you consider?	Age and weight	0.0%	1.0%	0.16
	Duration of treatment	10.5%	10.8%	
	Estimated child size	2.9%	1.0%	
	Medical history	0.6%	1.0%	
	One cartridge is given for all patients	5.8%	6.9%	
	Patient’s medical status	0.0%	1.0%	
	Precise age in years	5.8%	13.7%	
	Precise body weight	58.1%	56.9%	
	The level of difficulty of the procedure	15.7%	7.8%	
	Weight and child size and level of difficulty	0.6%	0.0%	
What needle gauge do you use most of the time for infiltration?	25 gauge	33.7%	38.2%	0.64
	27 gauge	45.3%	48.0%	
	30 gauge	19.2%	13.7%	
	Other	1.8%	0.0%	
What needle gauge do you use most of the time for blocks?	25 gauge	19.8%	25.5%	0.26
	27 gauge	68.0%	56.9%	
	30 gauge	10.5%	17.6%	
	Other	1.8%	0.0%	
What length of needle do you use most of the time for infiltration?	Long	2.3%	3.9%	0.22
	Short	95.3%	90.2%	
	Ultrashort	2.3%	5.9%	
What length of needle do you use most of the time for blocks?	Long	88.4%	74.5%	0.006 *
	Short	11.0%	25.5%	
	Ultrashort	0.6%	0.0%	
How long does it take you to inject a full cartridge of a local anesthetic solution?	10 s or less	9.3%	7.8%	0.87
	11–20 s	31.4%	31.4%	
	21–30 s	27.3%	23.5%	
	31–60 s	25.0%	30.4%	
	>60 s	7.0%	6.9%	

* Significant.

**Table 3:** Perceptions of local anesthetics among Saudi dental practitioners based on occupation.

Questions	Responses	Consultants	General Dentists	Specialist	*p*-Value
What kind of local anesthesia do you most commonly use?	Articaine	0.0%	1.7%	0.0%	0.81
	Bupivacaine	0.0%	0.4%	2.8%	
	Lidocaine	100.0%	91.7%	94.4%	
	Mepivacaine	0.0%	4.8%	2.8%	
	Prilocaine	0.0%	1.3%	0.0%	
When deciding on the dose of the local anesthetic you administer, what is the most important thing you consider?	Age and weight	0.0%	0.4%	0.0%	0.12
	Duration of treatment	12.5%	10.4%	11.1%	
	Estimated child size	0.0%	2.6%	0.0%	
	Medical history	0.0%	.4%	2.8%	
	One cartridge is given for all patients	0.0%	5.7%	11.1%	
	Patient’s medical status	0.0%	0.4%	0.0%	
	Precise age in years	50.0%	8.3%	2.8%	
	Precise body weight	25.0%	58.3%	61.1%	
	The level of difficulty of the procedure	12.5%	13.0%	11.1%	
	Weight and child size and level of difficulty	0.0%	0.4%	0.0%	
What needle gauge do you use most of the time for? (infiltration)	25 gauge	62.5%	35.7%	27.8%	0.01
	27 gauge	0.0%	47.4%	50.0%	
	30 gauge	37.5%	16.5%	16.7%	
	Other	0.0	0.4	5.6	
What needle gauge do you use most of the time for? (blocks)	25 gauge	62.5%	21.3%	16.7%	0.00 *
	27 gauge	0.0%	66.5%	61.1%	
	30 gauge	37.5%	11.7%	16.7%	
	Other	0.0	0.4	5.6	
What length of needle do you use most of the time for? (infiltration)	Long	25.0%	2.6%	0.0%	0.00 *
	Short	50.0%	93.9%	100.0%	
	Ultrashort	25.0%	3.5%	0.0%	
What length of needle do you use most of the time for? (blocks)	Long	87.5%	87.4%	55.6%	0.00 *
	Short	12.5%	12.6%	41.7%	
	Ultrashort	0.0%	0.0%	2.8%	
How long does it take you to inject a full cartridge of a local anesthetic solution?	10 s or less	37.5%	7.4%	11.1%	0.00 *
	11–20 s	0.0%	34.3%	19.4%	
	21–30 s	25.0%	27.8%	13.9%	
	31–60 s	12.5%	25.2%	41.7%	
	61 s or more	25.0%	5.2%	13.9%	

* Significant.

**Table 4:** Overall scores achieved by all participants regarding topical anesthetics used for children.

Questions	Responses
How often do you use a topical anesthetic (gel, spray, liquid, etc.)?	
Always	65.7%
Never	1.1%
Rarely	6.6%
Sometimes	26.6%
What form of topical anesthetic do you commonly use?	
Gel	88.3%
Liquid	1.8%
Ointment	0.7%
spray	9.1%
Patch	0.0%
What kind of topical anesthetic agent do you commonly use?	
Benzocaine	68.2%
Compounded topical anesthetics	12.4%
Lidocaine	19.3%
What brand of topical anesthetic do you use?	
Avocaine spray	0.4%
Benzojel	0.4%
Gingicaine (gel, liquid, spray)	4.4%
Hurricaine (gel, liquid, spray)	14.2%
I do not know	55.8%
Topex (gel, spray)	4.4%
Topicale (gel, ointment, patch)	20.4%
Would you like to use a different topical anesthesia delivery system if available on the market?	
Maybe	50.0%
No	12.4%
Yes	37.6%
How long do you wait to inject after applying a topical anesthetic?	
10 s or less	16.4%
11–20 s	18.2%
21–30 s	17.9%
31–60 s	23.7%
61 s or more	23.7%
Do you think that topical anesthetics are effective when used before the injection of local anesthesia?	
Adequate	20.4%
Effective	38.7%
Ineffective	1.8%
Poor	5.1%
Very effective	33.9%
What do your patients most complain about regarding the topical anesthetic available on the market today?	
Burning sensation	0.8%
Color	5.5%
Consistency	3.6%
Never complain	4.9%
Smell	1.5%
Taste	83.6%
How many of your patients experienced any drug side-effect to the topical anesthetic preparation in the last year?	
0%	82.8%
1–3%	14.6%
4–6%	1.1%
More than 7%	1.5%
In your years of practice treating pediatric patients, approximately what percentage of the time do topical anesthetics work when applied prior to the local anesthetic injection?	
0%	4.7%
25	24.8%
50%	22.3%
75%	36.1%
100%	12.0%

**Table 5:** Perceptions of topical anesthetics among Saudi dental practitioners based on gender.

Questions	Responses	Female	Male	*p*-Value
How often do you use a topical anesthetic (gel, spray, liquid, etc.)?	Always	68.6%	60.8%	0.22
	Never	1.7%	0.0%	
	Rarely	6.4%	6.9%	
	Sometimes	23.3%	32.4%	
What form of topical anesthetic do you commonly use?	Gel	89.5%	86.3%	0.86
	Liquid	1.7%	2.0%	
	Ointment	0.6%	1.0%	
	Spray	8.1%	10.8%	
	Gel	89.5%	86.3%	
What kind of topical anesthetic agent do you commonly use?	Benzocaine	68.6%	67.6%	0.21
	Compounded topical anesthetics	14.5%	8.8%	
	Lidocaine	16.9%	23.5%	
What brand of topical anesthetic do you use?	Avocaine spray	0.0%	1.0%	0.61
	Benzojel	0.0%	1.0%	
	Gingicaine (gel, liquid, spray)	4.1%	4.9%	
	Hurricaine (gel, liquid, spray)	15.1%	12.7%	
	I do not know	57.6%	52.9%	
	Topex (gel, spray)	4.1%	4.9%	
	Topicale (gel, ointment, patch)	19.2%	22.5%	
Would you like to use a different topical anesthesia delivery system if available on the market?	Maybe	52.3%	46.1%	0.38
	No	10.5%	15.7%	
	Yes	37.2%	38.2%	
How long do you wait to inject after applying a topical anesthetic?	10 s or less	19.8%	10.8%	0.09
	11–20 s	20.3%	14.7%	
	21–30 s	16.3%	20.6%	
	31–60 s	23.8%	23.5%	
	61 s or more	19.8%	30.4%	
Do you think that topical anesthetics are effective when used before the injection of local anesthesia?	Adequate	20.9%	19.6%	0.55
	Effective	41.3%	34.3%	
	Ineffective	2.3%	1.0%	
	Poor	4.7%	5.9%	
	Very effective	30.8%	39.2%	
What do your patients most complain about regarding the topical anesthetic available on the market today?	Burning sensation	0.6%	1.0%	0.14
	Color	2.9%	9.8%	
	Consistency	2.9%	4.9%	
	Never complain	4.7%	5.0%	
	Smell	1.2%	2.0%	
	Taste	87.8%	76.5%	
How many of your patients experienced any drug side-effect to the topical anesthetic preparation in the last year?	0%	86.0%	77.5%	0.28
	1–3%	12.2%	18.6%	
	4–6%	0.6%	2.0%	
	More than 7%	1.2%	2.0%	
In your years of practice treating pediatric patients, approximately what percentage of the time do topical anesthetics work when applied prior to the local anesthetic injection?	0%	5.8%	2.9%	0.89
	25%	28.5%	18.6%	
	50%	22.1%	22.5%	
	75%	34.9%	38.2%	
	100%	8.7%	17.6%	

**Table 6:** Perceptions of topical anesthetics among Saudi dental practitioners based on occupation.

Questions	Responses	Consultant	General Dentist	Specialist	*p*-Value
How often do you use a topical anesthetic (gel, spray, liquid, etc.)?	Always	62.5%	65.2%	69.4%	0.00 *
	Never	25.0%	0.0%	2.8%	
	Rarely	0.0%	7.4%	2.8%	
	Sometimes	12.5%	27.4%	25.0%	
What form of topical anesthetic do you commonly use?	Gel	100.0%	89.1%	80.6%	0.02 *
	Liquid	0.0%	2.2%	0.0%	
	Ointment	0.0%	0.9%	0.0%	
	Spray	0.0%	7.8%	19.4%	
	Patch	0.0%	0.0%	0.0%	
What kind of topical anesthetic agent do you commonly use?	Benzocaine	62.5%	68.3%	69.4%	0.13
	Compounded topical anesthetics	12.5%	13.0%	8.3%	
	Lidocaine	25.0%	18.7%	22.2%	
What brand of topical anesthetic do you use?	Avocaine spray	0.0%	0.4%	0.0%	0.01 *
	benzojel	0.0%	0.4%	0.0%	
	Gingicaine (gel, liquid, spray)	0.0%	4.3%	5.6%	
	Hurricaine (gel, liquid, spray)	50.0%	14.3%	5.6%	
	I do not know	0.0%	57.4%	58.3%	
	Topex (gel, spray)	0.0%	3.9%	8.3%	
	Topicale (gel, ointment, patch)	50.0%	19.1%	22.2%	
Would you like to use a different topical anesthesia delivery system if available on the market?	Maybe	0.0%	53.5%	38.9%	0.01 *
	No	0.0%	12.2%	16.7%	
	Yes	100.0%	34.3%	44.4%	
How long do you wait to inject after applying a topical anesthetic?	10 s or less	0.0%	17.8%	11.1%	0.00 *
	11–20 s	0.0%	21.3%	2.8%	
	21–30 s	62.5%	15.2%	25.0%	
	31–60 s	12.5%	22.6%	33.3%	
	61 s or more	25.0%	23.0%	27.8%	
Do you think that topical anesthetics are effective when used before the injection of local anesthesia?	Adequate	0.0%	22.2%	13.9%	0.00 *
	Effective	25.0%	40.4%	30.6%	
	Ineffective	0.0%	0.9%	8.3%	
	Poor	0.0%	3.9%	13.9%	
	Very effective	75.0%	32.6%	33.3%	
What do your patients most complain about regarding the topical anesthetic available in the market today?	Burning sensation	12.5%	0.4%	0.0%	0.35
	Color	0.0%	5.7%	5.6%	
	Consistency	0.0%	3.9%	2.8%	
	Never complain	0.0%	5.6%	5.6%	
	Sensation	0.0%	0.4%	0.0%	
	Smell	0.0%	1.3%	2.8%	
	Taste	87.5%	83.5%	83.3%	
How many of your patients experienced any drug side-effect to the topical anesthetic preparation in the last year?	0%	50.0%	83.0%	88.9%	0.00 *
	1–3%	25.0%	15.7%	5.6%	
	4–6%	0.0%	0.4%	5.6%	
	More than 7%	25.0%	0.9%	0.0%	
In your years of practice treating pediatric patients, approximately what percentage of the time do topical anesthetics work when applied prior to the local anesthetic injection?	0%	0.0%	4.8%	5.6%	0.00 *
	25%	0.0%	24.3%	33.3%	
	50%	50.0%	23.0%	11.1%	
	75%	25.0%	36.5%	36.1%	
	100%	25.0%	11.3%	13.9%	

* Significant.

## Data Availability

The data that support the findings of this study are available from the corresponding author upon reasonable request.
